# MAC Protocols for mmWave Communication: A Comparative Survey †

**DOI:** 10.3390/s22103853

**Published:** 2022-05-19

**Authors:** Pulok Tarafder, Wooyeol Choi

**Affiliations:** Department of Computer Engineering, Chosun University, Gwangju 61452, Korea; pulok@chosun.kr

**Keywords:** MAC protocols, millimeter-wave, resource allocation, scheduling

## Abstract

With the increase in the number of connected devices, to facilitate more users with high-speed transfer rate and enormous bandwidth, millimeter-wave (mmWave) technology has become one of the promising research sectors in both industry and academia. Owing to the advancements in 5G communication, traditional physical (PHY) layer-based solutions are becoming obsolete. Resource allocation, interference management, anti-blockage, and deafness are crucial problems needing resolution for designing modern mmWave communication network architectures. Consequently, comparatively new approaches such as medium access control (MAC) protocol-based utilization can help meet the advancement requirements. A MAC layer accesses channels and prepares the data frames for transmission to all connected devices, which is even more significant in very high frequency bands, i.e., in the mmWave spectrum. Moreover, different MAC protocols have their unique limitations and characteristics. In this survey, to deal with the above challenges and address the limitations revolving around the MAC layers of mmWave communication systems, we investigated the existing state-of-the-art MAC protocols, related surveys, and solutions available for mmWave frequency. Moreover, we performed a categorized qualitative comparison of the state-of-the-art protocols and finally examined the probable approaches to alleviate the critical challenges in future research.

## 1. Introduction

With the rapid increase in large-volume data sharing and cloud storage, high bandwidth communication is urgently needed. According to the standards issued by the International Telecommunication Union (ITU), a next-generation 5G communication network structure must jointly support the following three services: enhanced mobile broadband (eMBB), massive machine-type communications (mMTC), and ultra-reliable and low-latency communications (URLLC). Here, eMBB refers to the high bandwidth internet connection used in smartphones, mMTC focuses on the narrowband internet access mainly used in sensing and smart cities, and lastly, URLLC aims to achieve the lowest possible delay as low as 1 ms for sensitive applications [1].

Mobile wireless communication has progressed with current modern technologies that are capable of providing high-quality mobile broadband services at end-user data rates of several megabits per second over large areas and to tens of thousands of users. To meet the increasing demand for faster and more reliable wireless networks, mmWave frequency band is a prime choice for a modern 5G communication architecture. Based on the recommendations of the ITU, governments all over the world have sanctioned mmWave bands for commercial use. Although the mmWave bands operate between 30 and 300 GHz, corresponding to the wavelengths from 10 mm to 1 mm, 1.8 and 2.6 GHz bands are also incorporated with 5G [2].

As a result, the mmWave spectrum still has a large-scale unlicensed bandwidth. Due to the fundamental contrasts between mmWave communications and existing microwave-based communication technologies (e.g., 2.4 GHz and 5 GHz), mmWaves, by nature, demonstrate high signal attenuation. This fading channel phenomenon is expected to be frequency-selective [3]. A common solution of this problem is to introduce beamforming. Previously, mmWaves were considered inefficient by the researchers for mobile access networks because of their high vulnerability to shadowing and poor isotropic propagation loss [4]. However, even a small portion of the accessible mmWave spectrum can achieve hundreds of times the data throughput, and capacity realized by the existing cellular spectrum [5].

When facilitating the growing demands of mobile cellular devices, wearable sensors, and the Internet of Things (IoT) with this abundant mmWave spectrum, in next-generation communication systems (beyond 5G), where each of the devices will be incorporated with artificial intelligence (AI), coordination among the connected dense networks will be crucial. Additionally, with billions of connected devices, data transmission will create a long overhead. These issues can be carefully administered at the MAC layer [6], and this could lead to addressing the above-mentioned challenges in terms of access and networking point of view. Furthermore, in 5G networks, neighbor discovery and user coordination are fundamental aspects, which can also be dealt with at the MAC layer [7]. Moreover, the special propagation features and hardware requirements of mmWave systems described in [8,9] highlight numerous challenges for the MAC, PHY layer, and routing layers [10]. By synchronizing omnidirectional and directional transmissions, MAC layer protocols can significantly increase the overall efficiency of mmWave systems [11].

To circumvent the physical restrictions of mmWaves, and to facilitate the development of a smart communication system, advanced MAC protocols may need to be utilized simultaneously, specific to mmWave bands, and multiple communication layers with different coverage may have to be allowed to coexist [12]. Considering the above, to provide a better grasp of the possible implementation of the currently available MAC protocol and their feasibility for future mmWave wireless systems and pave the route for further development, the contributions of our survey are as follows.

**Figure 1:**
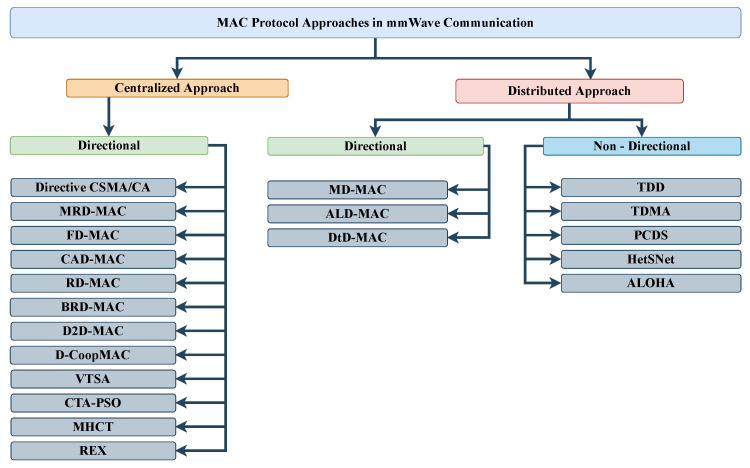
Structural classification of MAC protocols.

- In this survey, we investigated the current trends of different directional and non-directional MAC protocols, which have a strong potential for improving mmWave communication systems.
- We compared the existing surveys chronologically according to their publishing dates, identifying the research focus areas over the years, which is presented in Section 2.
- We classified all mmWave spectrum operating MAC protocols based on their methodologies into standard centralized and distributed protocols, which is illustrated in Figure 1.
- In this survey, we further investigated all the protocols and pointed out their scopes, limitations, and advantages. We summarize the centralized protocols and the distributed protocols in Section 4 and Section 5, respectively.
- We also identified the challenges regarding the design methodologies for the MAC protocols and pointed out the future research directions in the MAC layer domain of mmWave communication systems.
- Although there are a few surveys in the field of mmWave MAC issues, none of them have organized all available protocols with detailed review and classified them into standard categories, stating their descriptive advantages and disadvantages. Moreover, we noted the probable implementations for respective protocols in the comparative analysis table.

## 2. Existing Surveys

In recent years, there have been several advancements in the MAC layer, matched by the progress in the available MAC protocols that operate at mmWave frequencies, as shown in Table 1. Niu et al. [13] primarily studied WPAN related MAC protocols. In particular, they examined the IEEE 802.11ad and IEEE 802.15.3c standards for the 60 GHz band focusing on carrier sense multiple access/collision avoidance (CSMA/CA), memory-guided directional MAC (MD-MAC), and time division multiple access (TDMA) protocols. These protocols consist of a contention access period (CAP), channel time allocation period, piconet controller (PNC), and beacon period. Moreover, a significant amount of their work involves device to device (D2D) communication, PHY layer techniques, and wireless backhaul.

Gupta et al. presented a study of an interference management from a multi-input multi-output (MIMO) perspective, and described in general 5G network structures and partially described evolution of wireless technologies from a MAC perspective in [2]. Ghadikolaei et al. [10] conducted a supplementary study and addressed MAC layer issues in mmWave cellular networks, such as initial access, mobility management, resource block channelization, and different directional control mechanisms in the PHY layer of a mmWave band. In contrast, Agiwal et al. [14] addressed various multiplexing techniques, and mmWave based PHY layer aspects, and reviewed the changes required in the MAC layer of a mmWave system to support its PHY layer adjustments. Shokri-Ghadikolaei et al. [15] pointed out other common but important mmWave communication MAC design issues. Furthermore, they highlighted various IEEE standards used in the MAC layer of mmWave communication and addressed the collision and deafness problems of CSMA, TDMA, and ALOHA protocols.

In [16], Kim studied directional mmWave wireless systems for next-generation MAC approaches. In addition, Zhou et al. in [17] addressed MAC issues of various IEEE standards, channel access over multiple channels, interference mitigation, and reviewed the cross-layers between PHY and MAC layers. In [18], a compilation of the mmWave communications MAC protocols and scheduling systems for ad hoc networks, mesh networks, WPANs, and cellular networks from the literature were presented. They categorized their efforts into four areas; PHY layer, MAC layer, network layer, and cross-layer optimization. Furthermore, in [19], Han et al. conducted an in-depth study on MAC protocols for both mmWave and THz bands wireless networks. They also numerically analyzed the delay, network throughput, outage probability, and fairness index with varying node density.

Similarly, Mamadou et al. investigated existing communication protocols, strategies, and mechanisms, as well as 5G communication standard elements that help management of wireless technology with cohabitation [20]. They concentrate on access layer solutions for unlicensed frequency bands. Moreover, they also believe that resource sharing should be expanded to include not only spectrum management but also physical system management.

Uwaechia et al. [21] mostly investigated the fundamentals of MIMO and mmWave, and subsequently analyzed some multiple access protocols for 5G wireless networks. Finally, in [22], we illustrated a simplified overview of mmWave protocols, focusing only on centralized MAC protocols as the primary part of this survey.

**Table 1:** Comparative analysis of the existing surveys.

Ref.	Year	Title	Scope
[13]	2015	A Survey of Millimeter Wave Communications (mmWave) for 5G: Opportunities and Challenges	Interference management, spatial reuse, anti-blockage, and mobility dynamics for 60 GHz band
[2]	2015	A Survey of 5G Network: Architecture and Emerging Technologies	Distributed spectrum sharing and management, interference management, joint scheduling, and the procedure of various interference management in 5G based MIMO
[10]	2015	Millimeter Wave Cellular Networks: A MAC Layer Perspective	Scheduling, association, random access, synchronization, and interference management
[14]	2016	Next Generation 5G Wireless Networks: A Comprehensive Survey	Directional MAC protocols and their multiplexing techniques, mmWave based physical layer aspect
[15]	2016	Design Aspects of Short-range Millimeter-wave Networks: A MAC Layer Perspective	Short-range mmWave collision-aware hybrid resource allocation and multi-hop connectivity issues
[16]	2016	Millimeter-Wave (mmWave) Medium Access Control: A Survey	Directional mmWave beam management, relaying, and scheduling
[17]	2018	IEEE 802.11ay-Based mmWave WLANs: Design Challenges and Solutions	MAC issues for the 802.11ay, channel allocation, spatial sharing, and interference management
[18]	2018	Millimeter Wave Communication: A Comprehensive Survey	MAC protocols and scheduling systems for ad hoc networks, mesh networks, and wireless personal area networks (WPANs)
[19]	2019	On Medium Access Control Schemes for Wireless Networks in the Millimeter-wave and Terahertz Bands	Deafness issues, control channel selection mechanisms, blockage issues, mobility management, and spatial reuse strategies
[20]	2020	Survey on Wireless Networks Coexistence: Resource Sharing in the 5G Era	Resource sharing and access layer solutions
[21]	2020	A Comprehensive Survey on Millimeter Wave Communications for Fifth-Generation Wireless Networks: Feasibility and Challenges	Initial access, multi-hop overhead issues, mobility management, and handover
Our survey		MAC Protocols for mmWave Communication: A Comparative Survey	Resource allocation, interference management, anti-blockage, deafness issues, scheduling, association, random access and synchronization, multi-hop connectivity, and individual protocol review

## 3. MAC Layer and Beamforming for mmWave

### 3.1. MAC Layer

The 5G mmWave MAC layer will eventually be significantly redesigned to take advantage of the highly directed, ultra-low latency end-to-end service demands, as well as 20 times higher peak speeds than 4G [4,15,23,24,25,26,27,28]. In the ultra-dense mmWave networks, variations in traffic can occur faster than routes can be adjusted. Therefore, resource assignments inside a MAC layer must work in a faster time frame and be reactive to the immediately offered load. A routing layer assists the MAC layer for this purpose by providing link-specific information that classifies dedicated, restricted, and shared resources [29]. Moreover, MAC layers can be modified to support high streaming and downloading services in the ultra-dense mmWave networks and mmWave WPANs, which are considered to be the foundation of future generation communication systems.

However, there are several problems that need to be considered when providing mmWave services using the existing MAC protocols. Mobility management and initial access are some examples. These functions define how a user equipment (UE) connects and maintains its connection to the connected network. However, UEs must decode a shared directed channel so that they can retrieve system information in a mmWave cellular network. The network operating environment may also influence the MAC protocols that are best suited for deciding which MAC is the most appropriate for a case [30]. Scheduling based on only a partial understanding of the network architecture results in a considerable reduction in network throughput, around 33% as documented in [31]. However, discovering the topology (even partial information) necessitates the exchange of multiple control messages. Due to the uniqueness of the physical control channel in a mmWave network, the transmission of these control messages can be overwhelming [10]. In most cases, the physical control channel has a substantially reduced transmission rate as opposed to the data channel, owing to the increased resilience and robustness [30].

### 3.2. Beamforming

Mobile devices are highly prone to experience interference, and by nature mmWaves experience high blockage which significantly impacts the spectrum sharing and accessing. To overcome these interference and blockage issues, it is necessary to search for an alternate channel that is directed, and not blocked. Directed communication, referred to as beamforming, is the most widely used scheme in mmWave as discussed in Section 1. Moreover, beamforming requires continuous switching and scheduling, and transmission and reception beam operation can be regulated on the MAC layer [32]. In general, to support a densely populated indoor WPAN with more users, indoor concurrent beamforming is very crucial.

**Figure 2:**
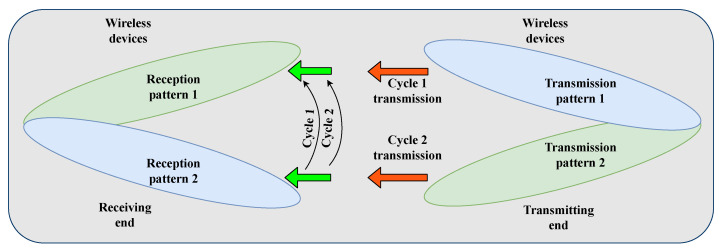
Single-link beamforming.

Concurrent beamforming protocols rely on receivers that detect the signal-to-interference plus noise ratio (SINR) [32]. Figure 2 presents a four-phase single-link beamforming scheme that can schedule the link transmission of wireless devices. Some mmWave MAC layer beamforming protocols can work efficiently by eliminating the need for the angle of departure (AOD) and angle of arrival (AOA) [33]. Moreover, it has been established that a MAC protocol that utilizes spatial properties could significantly boost the mmWave cellular network capacity [14]. Network architectures such as WPANs and wireless local area networks (WLANs) permit non-line of sight (NLoS) communication. However, the above-mentioned phenomenon complicates the cellular network architecture in terms of the mmWave MAC design [34].

From the base station (BS), the signals are propagated directly towards the users. In general, an MIMO antenna array is used to propagate a signal in a specific direction, and a beamforming protocol is required in the MAC layer of a system to choose the optimal transmission and reception beams based on the selection measure. In an indoor environment, in which the traffic is high because of numerous devices, beamforming can significantly reduce collisions among the signals and increase the quality of service (QoS) of the connected devices. However, the previously mentioned deafness problem is also prevalent in beamforming techniques, which are discussed in terms of MAC layer solutions in the next section.

### 3.3. Taxonomy of mmWave MAC Protocols

**Figure 3:**
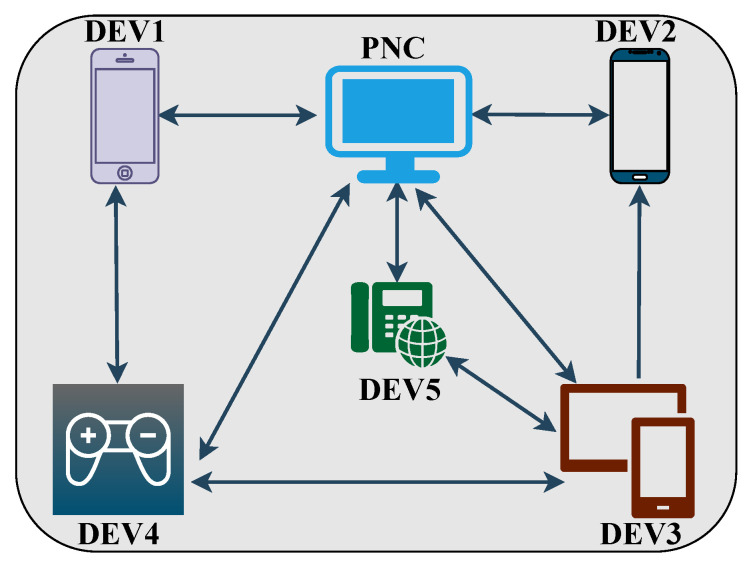
PNC and connecting devices for WPAN.

Traditionally, two approaches have been used for mmWave spectrum sharing and accessing [35]: centralized and distributed. Our survey classifies MAC protocols based on these two categories, as shown in Figure 1. A centralized approach is a refined and conservative approach in which a central unit called an access point (AP) or a PNC organizes the communication between the networks. Moreover, in the IEEE802.15.3c standard, a mmWave-WPAN can simply be called a piconet [36]. A PNC is used to centrally perform the channel assessment, power scheduling, and time slot distribution for the links. In contrast, in a distributed approach, the whole networks are coordinated with each other, and there is no mother-control AP or PNC. Figure 3 illustrates a PNC model with five connected devices.

## 4. Centralized Protocols

In this section, we briefly review the architectural design and characteristics of all current centralized MAC protocols that operate in the mmWave systems. Table 2 and Table 3 summarize the centralized protocols in terms of applications and comparative analysis, respectively.

### 4.1. Directive CSMA/CA

**Figure 4:**
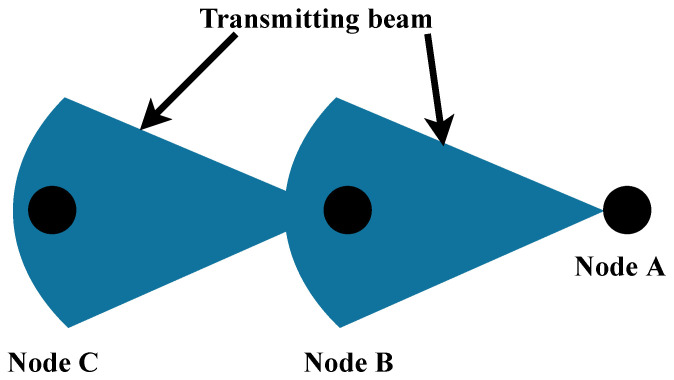
Deafness problem in directional beamforming.

Scheduling is a computation intensive approach in mmWave frequencies; consequently, implementing PNCs efficiently on a mobile device is arduous. IEEE 802.15.3 states that a PNC is required to arrange the bandwidth requests from linked devices. Legacy CSMA/CA protocols do not work well with the directional antennas because of the deafness problem in the wireless network nodes [37]. Here, when a transmission uplink and downlink is established between nodes A and C, node B is unable to sense the transmission because node A points towards node C. Thus, a deafness problem arises for directional beamforming at node B as shown in Figure 4.

To address this issue, a directional CSMA/CA-based MAC protocol was proposed in [37], in which the DEVs always maintain their positions by focusing their beams toward a PNC. This scenario is ensured before any communication link is established. The DEVs in the nodes dispatch a target request to be sent (TRTS) to the centralized PNC. Subsequently, upon finding a DEV that is ready to transmit a target clear to sent (TCTS), the PNC regulates the request accordingly and establishes a connection by overcoming the deafness problem.

**Figure 5:**
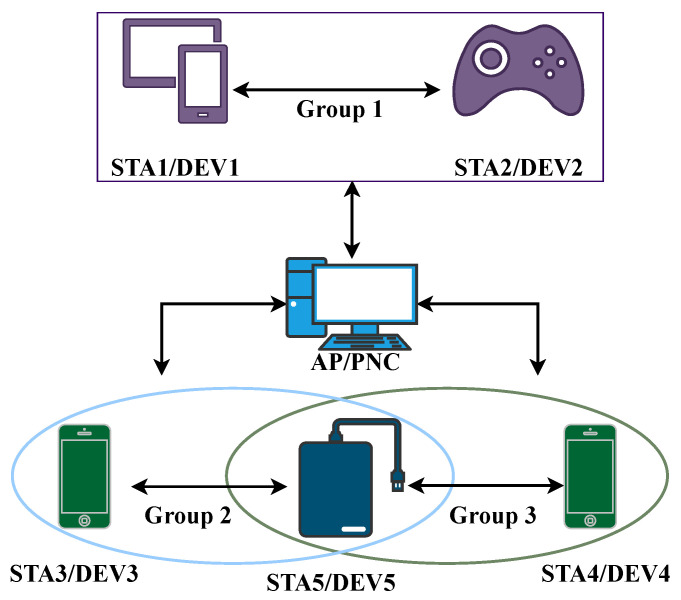
Operation of directive CSMA/CA.

A modified version of the above protocol [37] was presented in [38], utilizing spatial reuse by the same authors. In the revised scheme, the DEVs are called stations (STAs), and they form a peer-to-peer link. A few of the STAs are grouped together with an AP, which is basically the same as a PNC. An example is illustrated in Figure 5.

### 4.2. MRD-MAC

Multi-hop relay directional MAC protocol, which is abbreviated to MRD-MAC, is another directional MAC protocol for a 60 GHz WPAN, and was proposed in [39]. This approach is mostly suitable for multi-gigabit indoor mmWave WPANs equipped with electronically steerable directional antennas. Primarily, it is a combination of the traditional AP-based single-hop and multi-hop protocols in the nodes. The job of the single-hop mechanism is to retain the established mmWave links. Subsequently, with the help of PNC and sequential polling policy, the transmission links take place. The PNC always finds an alternative route to the next node if there is a failure on any particular node. The multi-hop mode is responsible for the relay action with other intermediate nodes. However, the overall system performance degrades owing to the lack of spatial reuse and burst traffic contestation on the single route transmission. The overall process can be divided into five stages, discovery algorithm, the normal mode of operation, trailing control phase, lost node discovery and establishing a relay path, and achievable rates.

- Advantages: In the above-mentioned protocol, adaptive beamforming antennas are used for improving the directivity in 60 GHz communication. It introduces a cross layer representation, and the design aspects address the blockage vulnerability and directivity issues of mmWaves. In terms of obstacle handling and operation in an unobstructed environment, the network throughput remains reasonably consistent.
- Disadvantages: There is a direct relation between the control overhead and the node counts with multi-hop connectivity. For every superframe, the AP of the MRD-MAC protocol needs to check with every terminal whether the relay node is still connected to the current superframe, which increases the overhead.

### 4.3. FD-MAC

The challenge of efficient scheduling in a mmWave WPAN was investigated in [40]. The proposed frame-based scheduling directional MAC (FD-MAC) is a collision-free transmission scheme. In this system, a PNC is deployed along with several DEVs, thus making it a centralized MAC protocol. The nodes and DEVs in the network direct their beams toward the PNC when they are idle. Moreover, the PNC also decides and controls the scheduling by managing the traffic requests from the DEVs and the nodes. In a 60 GHz FD-MAC, the network operation time is divided into non-overlapping frames, which further include a scheduling phase. The non-overlapping intervals are divided in such a way where each interval have equal lengths. These steps are executed by the PNC.

**Figure 6:**
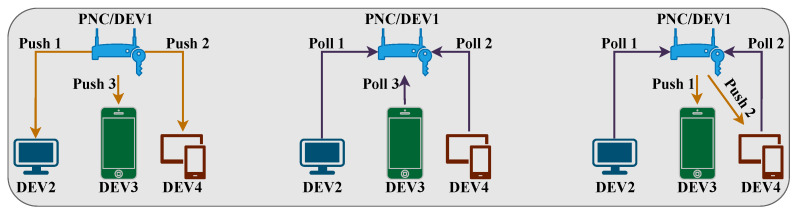
Architecture of FD-MAC protocol.

Specifically, each frame has scheduling and transmission phases. The operation of the above FD-MAC protocol is portrayed in Figure 6. A polling method [41] is employed in this protocol to correctly schedule, queue, and transmit packets. The PNC receives traffic patterns from DEVs and determines specific transmission schedules during the scheduling phase. Each schedule has sequences of topologies and time intervals, respectively. Afterwards, in the transmission phase, depending on the instructions of the scheduling phase, the DEVs perform their transmission. In addition, the PNC and the DEVs store the packets that arrive in the current time frame before re-transmitting them to the next frame.

- Advantages: As the frames are divided into two groups, the core scheduling algorithm of FD-MAC can compute a schedule for certain traffic requirements while maintaining overall transmission time. Virtual queue helps to schedule the backlogged packet arrival in the next frame. Moreover, the scheduling phase is dedicated to represent the overhead, hence no additional overhead is needed at the transmission phase.
- Disadvantages: Although the GC algorithm reduces the computation complexity, with the increase of network size, the execution time increases multiple fold when comparing a small network to a large network.

### 4.4. CAD-MAC

To address the limitations of traditional directional mmWave communication, such as providing coverage to only a specific region, there is another directional-based MAC protocol for mmWave WPANs, which is called coverage adaptive directional medium access control (CAD-MAC) [42] and is a nearly obsolete approach in the mmWave domain. The primary interest in this protocol is its usages of the 60 GHz band. The CAD-MAC executes two stages in order.

At first, sector management takes place, and adaptive regular S-CAP assignment is performed afterwards. The service range of the CAD-MAC is limited. In order to find out the appropriate beam width for transmission in the service range, sector management is conducted. Deploying a PNC is crucial in this role, because it collects the states of the DEVs connected in the network, including the distance in the individual sector, DEV ID, and area sector number. Another goal of using a PNC is to reduce the sector counts. From the achieved data, it analyzes the distances among the devices and the sectors are combined. Moreover, to execute these stages, the PNC also evaluates the exact antenna coverage range. Subsequently, adaptive regular S-CAP assignment aims to solve the discrepancy in the number of available devices in the network and delay. The role of PNC is again required at this stage. It counts the number of DEVs available in each sector and uses it to determine a regular S-CAP. Subsequently, in every sector, a minimum dynamic contention window is appointed.

- Advantages: The CAD-MAC protocol can deliver higher energy efficiency and throughput than the existing general protocols because it covers a larger service area with fewer sectors. In addition, the distance measurements in the sector management stage are performed by using received signal strength indication (RSSI), which is very reliable.
- Disadvantages: To cover all devices in the confinement, the CAD-MAC has to maintain a large number of sectors, and this can lead to degraded throughput performance. The nodes in a single sector do not ensure the same performance throughout the sector. Devices residing in the same sector can exhibit inconsistent network performance based on their distance and position in the sector.

### 4.5. RD-MAC

To address the challenges of optimum channel transmission rate measurement scheduling as a mixed integer linear programming (MILP), Niu et al. proposed a rate aware directional MAC (RD-MAC) in [43] for 60 GHz directional mmWave infrastructure. The RD-MAC consists of a central node and other general nodes. The central nodes are selected in such a way where they can maintain direct line-of-sight (LoS) with the other nodes and can keep all the clocks synchronized. The general nodes are called common nodes. Beamforming plays a crucial role in the network for steering because each node has to propagate its antenna array toward another node.

In the protocol, the frames are allocated to different time slots to ensure they do not overlap with each other. Each frame consists of two stages. The first step, known as the measurement stage, consists of common nodes steering their beams pointing in the direction of the center node. After pointing, the central node classifies and polls the common nodes in order based on the traffic demand vector of individual nodes. The central node subsequently responds to the common nodes depending on the traffic demand vectors, which is time-consuming. The traffic demand matrix is derived afterwards from the traffic demand vector data. Using the matrix, the central node creates a channel allocation measurement schedule. To relay these scheduling and allocation properties to the common nodes, the central node will sequentially steer its beam towards the commonly available nodes. Afterwards, upon receiving the parameters, the nodes measure the transmission rate and provide it as feedback to the central node. Thus, the central node in the network calculates the overall channel transmission rate matrix.

In the second stage, the protocol analyzes the data obtained in the first stage and generates an actual transmission schedule. The time slot counts, overall duration, and scheduling instructions are broadcasted all over the network to the common nodes on this stage for subsequent and concurrent transmission frames.

- Advantages: Different from other MAC protocols, owing to the segmentation of the working principle of the RD-MAC protocol, it can support multiple concurrent scheduling links under the condition that each link satisfies the SINR conditions and the channel transmission rate.
- Disadvantages: In the RD-MAC protocol, scheduling is executed in pairing, and the paired time slots are more emphasized. If the SINR of each of the links does not match up, it can affect the overall scheduling. Moreover, the frame duration is calculated by considering multiple time constraints, which can introduce an extra delay.

### 4.6. BRD-MAC

Relaying is a great choice of approach for mmWave infrastructures experiencing blockage issues. In addition to the previously presented RD-MAC protocol, Ref. [44] presented a blockage robust and efficient directional MAC (BRD-MAC) protocol for 60 GHz based directional mmWave communication. Corresponding to the RD-MAC, the BRD-MAC also has one central node, and the remaining nodes are called common nodes. However, here the nodes are half-duplex, and they have directional antennas, which are electronically steerable. Similar to the RD-MAC, the time frames do not overlap with each other, and the central node keeps track of the clocks of all the nodes and maintains them to stay synchronized. Segmentation of an epoch into scheduling and transmission phases also exists in this protocol.

- Advantages: The BRD-MAC is a versatile MAC protocol. This protocol considers relay selection, which is one of the most important future mmWave applications, making it more advantageous compared to other traditional protocols. It outperforms some other related MAC protocols such as FD-MAC and RD-MAC under different traffic patterns and blockage rates, demonstrating minimum transmission delay. It is a perfect protocol to tackle heavy load applications.
- Disadvantages: The probability of blockage increases as the number of hops increase. Therefore, the protocol is limited to operating under only two-hops. Such limitations have significant effect on the dynamic environments. As a result, the locations of the nodes and the propagation environment significantly impact the efficacy of the protocol.

### 4.7. D2DMAC

Dense deployment of small cells in the 60 GHz mmWave networks is gaining popularity with the deployment of 5G networks. The device-to-device MAC (D2D-MAC) protocol [45] is a frame-based protocol which is suited perfectly for dense mmWave small cells, and it can perform radio access and backhaul operations simultaneously. In this protocol, a frame can be divided into two stages: scheduling and transmission phases. In the first stage, the nodes of the system steer their antennas to their respective AP. Subsequently, according to the traffic demand of each node, each AP node relays the traffic demand status to the central controller with the help of a backhaul link. The controller node manages and schedules the traffic of all the established links in the network.

Later, on the second stage, based on the allocated schedule instructions, the nodes and the APs exchange data packets and establish the traffic flow with each other. This stage is called the transmission stage. At this stage, nodes can establish multiple simultaneous links. Moreover, finding these possible and optimal transmission paths among the nodes and APs are also controlled by the central controller. The protocol highly emphasizes the direct transmission links between two nodes. In order to achieve the near-optimal performance of indirect transmission path planning, the protocol always considers a path containing high channel quality.

Meanwhile, Qiao et al. [46] also proposed another D2DMAC for the mmWave with a combination of 4G system architecture with a TDMA-based MAC structure as a possibility for 5G cellular networks, with the 4G system performing the control operations. Due to the large capacity of mmWave communications, traffic can be offloaded from macrocells and improved services can be provided for traffic with high throughput needs. Concurrently, handovers between the macrocells and AP BS in the mmWave band, on the other hand, can deal with issues such as blockage, mobility management, and load balancing.

- Advantages: The D2D-MAC is primarily focused on its optimal application in the mobile access network, making it an appropriate candidate for small cell cellular networks. It has an optimal path selection criterion between the multiple small cells. It reduces the number of time slots required to facilitate all traffic flows. In addition, the small cells have the features to provide high speed data linking at high frequencies within a small coverage.
- Disadvantages: In D2D communication, setting up a direct LoS link requires users to discover each other. Inter-inference between devices poses a significant challenge in neighbor discovery. In addition, mode selection and synchronization is still an issue which remains in D2D protocols and communication.

### 4.8. D-CoopMAC

The directional cooperative MAC, known as D-CoopMAC protocol [47], is a comparatively complicated mmWave MAC architecture that works with the help of either an AP or personal basis service set (PBSS) control point and obtains essential timing and the allocation properties of service periods. Traditional cooperative communication with omnidirectional antennas ensures that a two-hop link performs better than a single-hop link. In addition, D-CoopMAC makes use of a relay STA to transmit data to the target AP or the PBSS control point (PCP).

The overall strategy has two distinguishable approaches, named basic mode and cooperative mode. In the basic mode, the communication between the STA and PCP/AP occurs with only single-hop communication. On the contrary, cooperative mode takes advantage of request to send (RTS), clear to send (CTS), and two-hop communication. When the source is ready to transmit the data packets, it transmits an RTS packet. The packet then reaches the destination (PCP/AP) through a relay STA. In return, using that relay medium, if the transmission connection between the PCP/AP and the source is possible, and also if the PCP/AP is ready to reply to that transmission, it responds with a directional multigigabit CTS. Only after the confirmation, the two-hop link is established. After the successful connection, an acknowledgement (ACK) packet is exchanged between the PCP/AP and the source.

- Advantages: This protocol model occupies a directional antenna scheme where it has a quasi-omni antenna pattern with the broadest beam bandwidth (360°). Consequently, when two or more non-PCP/non-AP STAs transmit to the PCP/AP at the same time, the deafness problem is eliminated.
- Disadvantages: D-CoopMAC is unsuitable for mobile networks because it involves the collection of data gathering based on the previous data; hence, it cannot ensure optimum relay selection. Moreover, the protocol is only based on the consideration of uplink channel access.

### 4.9. VTSA

Researchers developed the virtual time-slot allocation (VTSA) to make use of mmWave’s free space path loss [6]. In the event of omnidirectional antennas, the VTSA technique can also be used. When established communication links are more than 1 m apart, the proposed approach can schedule time slots so that multiple links can use the same time slot and reuse space. As it is a centralized protocol, a PNC manages co-channel interference (CCI), which is generated by the sharing of the channel time allocation (CTA). As a consequence, the same CTA can most likely be reassigned to multiple long-distance connections. Furthermore, the PNC employs a probing signal broadcasting period (PSBP) method that includes headers to determine the possible CCI. However, the PSBP must employ modulation and coding techniques to evade header packet loss.

- Advantages: The VTSA is meant to allow several communication channels to reuse TDMA time slots simultaneously to enhance the system throughput, while also monitoring the potential performance degradation owing to co-channel interference, thus increasing the transmission efficiency.
- Disadvantages: Since the VTSA uses multiple simultaneous links and uses the same CTA, it can experience signal interference because of the sharing. In addition, it is impossible to ensure that each connection receives access to a single superframe.

**Table 2:** Applications of centralized MAC protocols.

Ref.	Protocol Name	Scenario	Application	Simulation Tool
[37,38]	Directive CSMA/CA	WPAN	Deafness and collision avoidance	OPNET Modeler
[39]	MRD-MAC	WPAN, indoor office	Contention free transmission, anti-blockage	MATLAB, QualNet
[40]	FD-MAC	WPAN	Scheduling	MATLAB, C
[42]	CAD-MAC	WPAN, indoor conference room	Network throughput, sector management	Not specified
[43]	RD-MAC	WPAN	Scheduling, channel transmission rate measurement	MATLAB
[44]	BRD-MAC	WPAN	Anti blockage, relay selection, scheduling	Not specified
[45]	D2D-MAC	Heterogeneous small cells, outdoor cellular	Backhaul, D2D, access	Not specified
[47]	D-CoopMAC	WLAN	Channel access, uplink	C++, SMPL
[6]	VTSA	WPAN	System throughput, allocation	MIRAI-SF
[48]	CTA-PSO	WPAN	Multimedia, internet protocol television (IPTV), resource allocation, video on demand (VoD)	Not specified
[49]	MHCT	WPAN, indoor office room	Relay selection, scheduling	C++
[50]	REX	WPAN, indoor conference room	Scheduling	C

### 4.10. CTA-PSO

Particle swarm optimization (PSO) is well-known for its ease of use and high efficiency. PSO is built on social behavior and generates several potential solutions to a problem caused at initialization. In the algorithm, a swarm is a collection of solutions, and each answer is a particle. The particles go across the problem search space in quest of the best solution. In [51], it is disclosed that the PSO algorithm is suitable to resolve the resource allocation problem in mmWave wireless multimedia networks.

The authors [48] presented how CTA-PSO can handle the resource allocation problem even when there is a blockage with a live IPTV. IPTV does not cache previous frames, hence when a user changes channels, the IPTV has difficulty meeting the delay limitations. There is also a blocking issue, which is possible to overcome by incorporating a switch relay into the system. Furthermore, CTA-PSO adapts to the relay very quickly, and thus, even though there is a direct LoS blocking, CTA is assigned continuously.

- Advantages: The CTA-PSO shows excellent performance and is known for its near-optimal solution. It can seemingly deal with resource allocation across multiple applications, overcoming the issue of assigning a fixed bandwidth across arbitrary devices. Therefore, the CTA-PSO protocol does not demand network resource planning in advance.
- Disadvantage: The implementation of PSO in CTA increases the complexities in the network. In addition, in dynamic environments, the overall scheme may need to run again because of the nature of PSO if there are any unprepared problems and the collection of solutions is not feasible.

### 4.11. MHCT

In mmWave 5G networks, owing to the overwhelming connections and traffic flow, and because of the signal attenuation over distance, multi-hop mechanisms are adopted to improve the flow throughput. A multi-hop concurrent transmission (MHCT) was introduced by Qiao et al. in [49] to address these issues. The authors presented a novel hop selection measure to select data relays and forward the data using the selected relays. Selecting shorter links ensures high data transmission data rates. Consequently, as the hop count increases, heavy traffic also becomes visible, and the need for an appropriate hop selection is critical for this scenario. Upon receiving transmission requests, a PNC assigns appropriate relay hops by accumulating the global network information, separation distance among neighboring nodes, antenna directions, and traffic. In addition, the PNC creates weighted graphs between nodes to facilitate relay hop assignment. The final concurrent transmission method is then implemented based on this assignment.

In a mmWave network topology, the PNC maintains track of all updates and changes. When there are traffic demands, the PNC estimates the mean connection length and the traffic load, depending upon the topology updates. Eventually, the PNC enumerates the lowest cumulative weights using the Dijkstra algorithm and finally assigns hop selection.

- Advantages: The MHCT protocol has enabled multi-gigabits-per-second transmission at the indoor mmWave WPANs. To maximize the flow throughput, multi-hop transmissions can be used to overcome the link outage problem and counter the extreme propagation loss at the mmWave band.
- Disadvantages: The number of hops used for each traffic flow is highly influenced by the network topology. The summation of the link length to power for each traffic flow decreases as the number of short hops for each traffic flow rises, whereas the summation of the node loads increases. Hence, congestion increases at the nodes as the number of hops increases.

### 4.12. REX

Concurrent transmissions in WPANs can outperform standard serial TDMA transmissions in mmWave networks. A randomized exclusive region (REX) was created primarily in response to concurrent transmission and to investigate spatial multiplexing and resource management concerns for mmWave WPANs [50]. If there are fewer interferences, the REX protocol can outperform the traditional serial TDMA transmissions. The number of active flow requests in the WPAN, which the PNC of the protocol is aware of, is first determined. The proposed algorithm chooses a flow at random that has the fewest number of slots available. Afterwards, the algorithm checks the remaining active flows to determine whether they satisfy the concurrent transmission requirements. Following that, the scheme allocates slots one by one and arranges the flows accordingly. This procedure is repeated until all flows are scheduled.

- Advantages: REX is one of the first protocols to address the resource management issues in mmWave WPANs, and its scheme execution is unexpectedly simple. It utilizes a concurrent transmission scheme and as a result it outperforms the traditional serial TDMA transmission.
- Disadvantages: In the protocol, the authors created their model based on the free space path loss. This free space path loss model, which calculates the interference level and the received signal strength, is not ideal for indoor WPANs, since signal reflections would also create interferences. Moreover, the computational complexity is high for the REX protocol.

**Table 3:** Comparative analysis of centralized MAC protocols.

Ref.	Protocol Name	Spatial Reuse	Anti-Blockage	Targeted Wavelength	Key Idea	Limitations
[37,38]	Directive CSMA/ CA	Suppor- ted	Not specified	60 GHz	Executing low overhead action in congested networks, utilizing Markov decision process (MDP) to achieve high throughput	No priority assignments in the nodes
[39]	MRD-MAC	Not specified	Suppor- ted	60 GHz	Multi-hoping feature enables robust linking	No consideration for concurrent connectivity
[40]	FD-MAC	Suppor- ted	Not supported	60 GHz	Low complexity, great fairness performance, considers psuedowired interference models	Antiblockage related solutions are unavailable
[42]	CAD-MAC	Not specified	Not specified	60 GHz	Exhibits high network throughput and energy efficiency	Performance at 60° and 90° beamwidth is poor, consumes high power, exhibits long delay
[43]	RD-MAC	Suppor- ted	Suppor- ted	60 GHz	Concurrent transmissions are exploited	No consideration of allocation capacity
[44]	BRD-MAC	Suppor- ted	Suppor- ted	60 GHz	Utilizing relay selection to overcome blockage	Maximum two-hops are allowed, and complex blockage might need more than two hop to ensure robust link
[45]	D2D-MAC	Suppor- ted	Suppor- ted	60 GHz	Achieving near optimal delay and throughput	NLoS transmission is not considered
[47]	D-Coop MAC	Not specified	Not specified	60 GHz	Creating two-hop links via relay station	No backhaul networking scheme was integrated
[6]	VTSA	Suppor- ted	Not specified	60 GHz	Maintaining low overhead and computational complexity	Optimization is not incorporated
[48]	CTA-PSO	Not specified	Suppor- ted	60 GHz	Efficiently distributing resources even when there is a blockage, reducing delay	High computational complexity
[49]	MHCT	Suppor- ted	Suppor- ted	60 GHz	No novel relay selection matrix is presented	Complex scheduling algorithm
[50]	REX	Suppor- ted	Not specified	60 GHz	Obtaining a significant spatial multiplexing gain	Owing to scheduling repeatations computational complexity increases with time

## 5. Distributed Protocols

In this section, we briefly discuss the architectural design and features of all current distributed MAC protocols that operate in the mmWave systems. Table 4 and Table 5 summarize the distributed protocols in terms of applications and comparative analysis, respectively.

### 5.1. HetSNet in mmWave

Heterogeneous and small cell networks (HetSNets) use hierarchical deployments to improve the spectrum efficiency and throughput in mmWave networks. Considering this, the authors in [52] proposed a new frame scheme on the basis of time division duplex, and the scheme is 3GPP backward compatible. The proposed method ensures backhaul links and high-capacity access.

Therefore, in future 5G cellular networks, a combination HetSNets with mmWave bands will play a critical role. However, there are numerous issues with deploying HetSNets with the mmWave band 5G networks. Even though there is abundant literature on the PHY layer [53], only a few published studies in the literature have addressed the communication difficulties from the perspective of access and networking [32].

With the integration of mmWave communications with the HetSNet, a variety of deployment opportunities involving mmWave communication of backhaul user access lines have emerged [54]. Macrocell eNB (MeNB) is a standard HetSNet component. There are also other components such as multiple small cell eNBs (SeNBs), which can be further divided into relay eNBs, picocells, and femtocells. Before forwarding data to the MeNB in such a network, a combination process takes place. The backhaul data of every SeNB combines with the received data from the other nodes which exist in the network. Short distances (approximately 100–200 m) are supposed to separate the SeNBs, which helps mitigate high propagation losses. A mmWave radio may also provide user access coverage within small cells by lowering the interference level, which can be observed on the typical sub-3 GHz frequency ranges.

**Figure 7:**
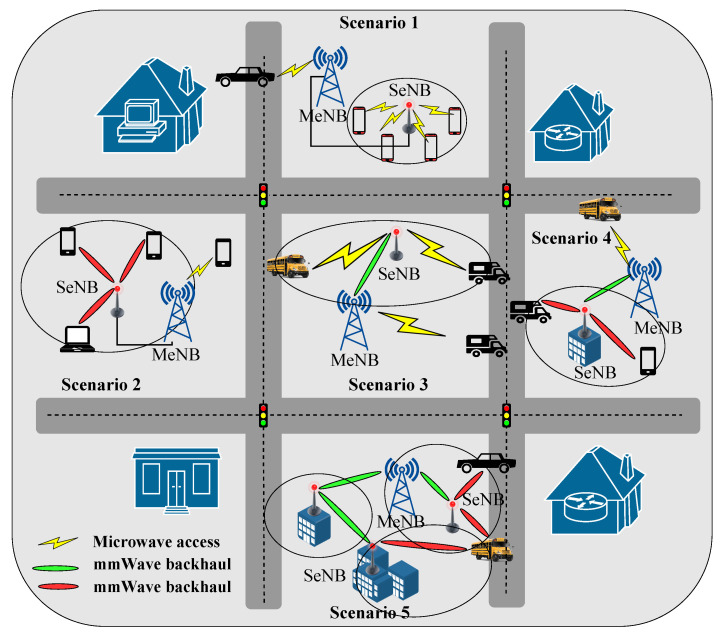
Architecture of HetSNet.

Figure 7 presents a traditional HetSNet architecture where several scenarios are illustrated. In scenario 1, an SeNB is connected to its donor MeNB through a wired backhaul. Moreover, UEs are served by both MeNB and SeNB. In scenario 2, UE’s communication with the MeNB takes place on the traditional microwave bands and with SeNBs, mmWave radio. In scenario 3, there is no wired backhaul where a single-hop mechanism is permitted for executing backhaul with mmWave band. For scenario 4, through single-hop mmMwave wireless backhaul executes the backhaul for the SeNBs, and the SeNBs serve the UEs with mmWaves. Finally, in the 5th scenario, dense small cells are deployed to connect multi-hop wireless backhaul between SeNBs and MeNB. Here, scenario 1 is the baseline, and scenarios 2–4 form a subset of scenario 5.

**Figure 8:**
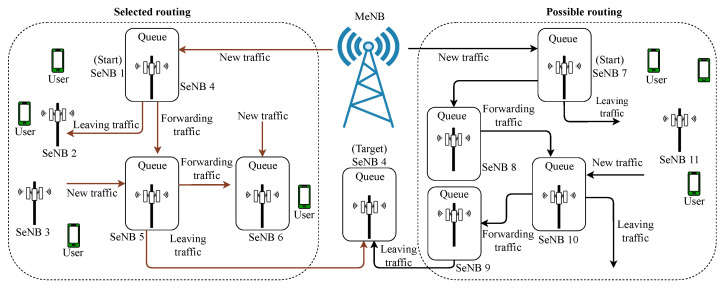
HetSNet routing operation.

Figure 8 represents a standard routing protocol scheme. It demonstrates an example of a possible path from a SeNB to the MeNB, as well as the tandem queue associated with it. Each queue may receive data traffic from a variety of sources. Traffic from connections other than the one being considered can enter and exit the tandem system at any time. By thoroughly searching all possible routes, the best practicable path can be discovered. This technique, on the other hand, has a substantial signaling overhead and prohibitively high computing complexity.

- Advantages: The HetSNet protocol implementation in the 3GPP standards enables us to overcome numerous key mmWave communication difficulties while also achieving an aggregated cell throughput of almost 13 Gb/s, which is an order of magnitude higher than that of the current best 5G system design [55].
- Disadvantages: Routing in the HetSNnet protocol can result in a substantial signaling overhead and a prohibitively high computing complexity. Even though the control overhead and delay may be reduced by applying a hierarchical routing scheme, however, the complexity still remains an open issue.

### 5.2. ALOHA

The ALOHA was first introduced in the 1970s [56], and almost every cellular network, including mmWave 5G network technology, uses this protocol [57]. ALOHA is a random access protocol in which various STAs transmit the data simultaneously, ignoring the collision realities. The advanced slotted ALOHA was introduced to address the issues present in the pure ALOHA. In slotted ALOHA, a time frame of transmission is divided into multiple discrete intervals, which are referred to as slots. Transmissions in slotted ALOHA are scheduled to commence at the beginning of each time slot. Since devices are synchronized using base STA synchronization signals, slotted ALOHA is an appropriate model for the worst-case analysis of a device-to-device (D2D) network underpinning a cellular network [58]. Furthermore, slotted ALOHA provides an upper bound for the throughput performance of pure ALOHA, in which transmission begins instantly upon the arrival of a new packet [30].

- Advantages: The fundamental benefit of ALOHA’s multi-hop context is that it seeks to send a packet as far as feasible in a mobile network. Due to the simplicity of ALOHA, it can be considered one of the most reliable wireless protocols for mobile communication systems.
- Disadvantages: Since the advanced version of aloha introduces a slotting mechanism, based on the scheduling necessities, few slots might stay idle during a transmission. This reduces the throughput of the network, causing the protocol to not perform at its peak occasionally. It also requires queue buffers for packet retransmission, and clock synchronization still remains an issue.

### 5.3. TDD & TDMA

Time division duplexing (TDD) and TDMA are some of the oldest traditional protocols that are still in use. The TDD protocol is commonly assumed to be the preferred mode of operation for 5G mmWave systems because it allows for better usage of broader bandwidths and the use of channel reciprocity for channel estimation [59,60]. The implementation of TDD/TDMA protocol in 60 and 73 GHz mmWave WPANs are presented in [61]. In a TDD system, time is divided between the users. The uplink and downlink transmissions are separated by a time interval in a synchronized manner. In this technique, uplink sounding signals can be used to collect the channel state information (CSI) at the transmitter in time division duplexing (TDD) systems.

However, the network’s performance may be limited by the limited UE power and the likely underdeveloped beamforming in the uplink communication reference signals. As a result, TDD in the mmWave frequencies must be limited to low-mobility circumstances. In the TDMA, several STAs share only one channel in entirely different time slots. The optimal spatial time division multiple access (STDMA) protocol utilizes some transceivers with minimal interference at a given resource block, providing the highest possible sum-rate for the whole network system [11,62,63,64].

Nonetheless, it requires a thorough understanding of network architecture [58], which is not supported by WPANs, particularly the devices in the move [30]. Time division multiple access (TDMA) reduces the overall overhead of STMDA. In TDMA protocol, every device is served one at a time. This sequencing ensures that no data is lost in the event of a collision.

- Advantages: From the implementation, it is noticeable that the TDD and the TDMA scheme is an outstanding candidate for mmWave WPANs. The use of TDD, particularly dynamic TDD, makes the 5G system incredibly flexible and bandwidth and power efficient. Moreover, the tiny subframe time slot length makes it easier to achieve the URLLC target latency (1 ms) [65].
- Disadvantages: In mmWave WPANs, multipath interference in a common circumstance. The use of TDD/TDMA can be substantially affected by this occurrence. For mobile phones, especially handhelds, TDMA on the uplink channel necessitates a high peak power in transmit mode, which reduces battery life. The TDMA also necessarily requires significant signal processing for matched filtering and correlation detection in order to synchronize with a time slot [66].

### 5.4. PCDS

Based on Zipf’s law, with the ever-growing increase in content delivery services (for instance Spotify, Netflix, Hulu), it is found that out of all available content on any streaming sites, only a small percentage of content accounts for a majority of the requests/streams over and over. In [67], the authors addresses this issue with a scheme called popular content downloading scheduling (PCDS) for the mmWave networks. It is an extended version of the previously discussed D2D [45] protocol.

In the PCDS protocol, the small cells in the mmWave architecture consist of one AP, and the others are users. Both the AP and users are rigged with steerable directional antennas. Thus, it is ensured that any two nodes can execute a directional propagation. In the 60 GHz wavelength, two approaches are used for neighbor discovery, named direct discovery and gossip-based discovery [68] for PCDS. The nodes can have a maximum of one established link with the surrounding nodes.

Popular contents are downloaded from the AP and are distributed to the users. This distributive operation operates sequentially. In the sequence, the scheduling and transmission phase mechanisms are similar to the other two-stage protocols such as the FD-MAC. Even the non-overlapping time dividing sequence is also maintained on PCDS. Furthermore, the packets or contents which the users download are sourced from the network layer.

- Advantages: The PCDS protocol incorporates a heuristic transmission path selection algorithm technique for establishing multi-hop transmission paths, which has excellent utilization of D2D communications and spatial reuse. Moreover, PCDS has outstandingly short delay and significant throughput performance according to numerous simulations.
- Disadvantages: Even though the overall performance is excellent, the computational complexity is high for the PCDS protocol. Also, adjacent lines cannot be scheduled for concurrent transmissions due to the half-duplex assumption in the PCDS protocol. Consequently, connections that share similar nodes cannot be scheduled together.

### 5.5. MD-MAC

For outdoor mesh networks in the 60 GHz mmWave bands, memory-guided directional MAC (MD-MAC) was proposed in [69]. Approaches similar to MD-MAC does not need any resource allocation or extensive cooperation among different nodes in the communication system. In this protocol, all the devices maintain their own allocation properties. They do not share their uplink and downlink state information with other nodes in the network. These properties and data are stored in an allocated slot, and the slot is kept updated after or before every single time frame. The state of a previously idle slot switches from idle to transmit following a successful transmission. Meanwhile, it also collects and saves its neighbor’s information. Jain’s fairness index (JFI) is frequently used to measure the fairness performance of the MD-MAC protocol. For node counts of 10, 20, and 30, the JFIs of the MD-MAC protocol are 0.90, 0.81, and 0.78, respectively.

- Advantages: The MD-MAC has the ability to quickly adapt and maintain link-level fairness. This memory-guided protocol is an excellent choice for outdoor mesh networks. Most of the mmWave MAC protocols do not take into account the deafness problem; however, the MD-MAC protocol uses predictability and learning to deal with this issue while demonstrating a small control overhead.
- Disadvantages: Based on the JFI, the MD-MAC protocol falls slightly behind some other commonly used MAC protocols because of the interferences. This could an explanation for why the MD-MAC is not suitable for indoor mmWave WPANs.

### 5.6. ALD-MAC

The neighbor discovery mechanism is a great choice for directional propagation systems, as it leads to the establishment of connections without redundancy transmission in random directions. Accordingly, a reinforcement learning (RL) based MAC protocol called adaptive learning directional medium access control (ALD-MAC) [70] was proposed to enable implicit cooperation between different nodes in the mmWave communication systems by combining a neighbor discovery algorithm with RL. In ALD-MAC, the channel access period is divided into a set of fixed-length frames. Each frame is further subdivided into a number of slots. Each node attempts to send a packet in the designated sector in each slot, and each slot’s duration is set to be long enough to deliver a packet with the maximum size. The JFI is also used to evaluate the performance index for ALD-MAC. For a node count of 10, 20, and 30 ALD-MAC has an index of 0.91, 0.87, and 0.80, respectively.

- Advantages: To gain a deeper knowledge of the network, ALD-MAC protocol provides implicit cooperation among diverse nodes. In addition, simulation results have demonstrated that ALD-MAC outperforms some traditional directional protocols, such as Directional Slotted ALOHA and MD-MAC.
- Disadvantages: RL is a powerful deployment tool for unknown environment applications, however, applying RL in real-life MAC applications has its drawbacks. Since the ALD-MAC protocol strongly depends on the neighbor discovery, RL might perform considerably poorly in discovering neighbors in the initial stage of the protocol execution because RL learns as it iterates in the environment by trial and error.

**Table 4:** Applications of distributed MAC protocols.

Ref.	Protocol Name	Scenario	Application	Simulation Tool
[69]	MD-MAC	Outdoor mesh networks	Deafness and interference reduction	QualNet
[70]	ALD-MAC	Not specified	Neighbor discovery	Not specified
[71]	DtD-MAC	Ad hoc networks	Deafness and collision avoidance	QualNet
[30,56,57]	ALOHA	Ad hoc networks, sensor networks, homogeneous mobile networks	Multi-hop selection	Not specified
[61,62,64]	TDD, TDMA	Ad hoc networks	Access, slot allocation	Ns-3
[52]	HetSNet	Heterogeneous small cells	Access, backhaul	Not specified
[67]	PCDS	Small cells	D2D, content delivery	QualNet

### 5.7. DtD-MAC

Traditionally, it is considered that the wireless nodes in a network can perform both omnidirectional and directional transmission. Nonetheless, a node is unable to sense multiple directions simultaneously; thus, this leads to the deafness problem and increases the chance of collision. Consequently, in 60 GHz ad hoc networks, establishing communication links with an accurate directional network allocation vector (DNAV) is not feasible.

Addressing these issues, Shihab et al. in [71] introduced a DtD-MAC for mmWave ad hoc networks. DtD-MAC stands for directional-to-directional (DtD) MAC protocol. In this architecture, the sending node collects the angle of arrival (AoA) of any existing incoming message. Concurrently, all nodes in the architecture also estimate the AoA of any incoming transmission. Afterwards, to predict the next-hop node’s location, the node analyzes the cache before any sensing or transmission occurs. Moreover, the idle nodes of the networks reduce the crucial directional deafness problem by continuously swapping their sensing directions, be it clockwise or anticlockwise. In addition, the protocol also ensures that the receiver receives the information. A node sends multiple directional RTS before receiving a CTS from the receiver.

- Advantages: The directional properties of the DtD-MAC enables both the senders and receivers to solve the asymmetry-in-gain problem. To capture the continually scanning idle receiver, the sender sends several DRTS packets to the receiver. These behaviors lead the protocol to mitigate the impact of the problems of deafness and collision of mmWave bands.
- Disadvantages: The primary aim of the DtD-MAC or neighbor discovery is associated with a disadvantage. Idle nodes must switch their sensing orientation either in clockwise or anticlockwise, and this makes it a power intensive protocol. During this process, extensive DRTS and CTS packets are exchanged, which may produce numerous handshake messages at the sender’s end, and limit the performance of the protocol as the beam count increases.

**Table 5:** Comparative analysis of distributed MAC protocols.

Ref.	Protocol Name	Spatial Reuse	Anti-Blockage	Targeted Wavelength	Key Idea	Limitations
[69]	MD-MAC	Not supported	Not specified	60 GHz	Using MDP guided predictability to address deafness problem	Poor performance when interferences are present
[70]	ALD-MAC	Not specified	Not specified	Not specified	Using ML algorithms for neighbor discovery	Computation hungry ML algorithms has limited performance over wireless nodes
[71]	DtD-MAC	Suppor- ted	Not specified	60 GHz	Overcoming asymmetry-in-gain problem	No arrangement for channel state characteristics
[30,56,57]	ALOHA	Not specified	Not specified	60 GHz	Making a transmission protocol into a basic and simple form	Completely discards the collision avoidance
[61,62,64]	TDD, TDMA	Not specified	Not specified	60 GHz, 73 GHz	Using a single channel to execute the networking	Only a limited number of users can be assigned to a single channel
[52]	HetSNet	Suppor- ted	Not specified	2 GHz, 28 GHz	Deployment of multi-hop routing	Requires large environment space for implementation, no efficient practical deployment data is available
[67]	PCDS	Suppor- ted	Not specified	60 GHz	Near optimal delay and throughput	Only prioritizes popular content over important and rare contents

## 6. Challenges & Future Research Directions

**Figure 9:**
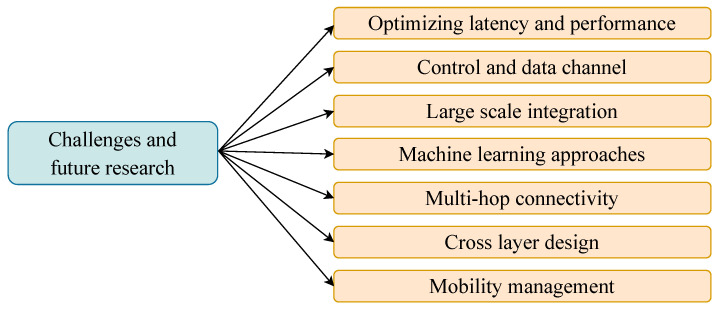
Challenges & future research directions.

Even though the centralized and distributed MAC protocols for mmWave communication have been extensively scrutinized, this research area still faces several challenges. This section highlights and points out some outstanding research questions and problems of mmWave MAC protocols, as shown in Figure 9.

### 6.1. Optimizing Latency and Performance

Next-generation 5G wireless networks differ significantly from conventional 3G/4G cellular systems, with more extreme performance and QoS requirements. Owing to the substantial open bandwidth in the mmWave spectrum, optimal channel modeling and resource allocation protocols are stringent. Until now, many multiplexing techniques have been implemented in wireless networks; however, to achieve low latency and high performance, further research is needed on the existing multiple access protocols. As PHY layer technologies approach the Shannon capacity [72], optimizing the existing MAC layer protocols and searching for new, fast, and efficient MAC protocols which ensures data privacy and device security when devices are connected to public WPANs, are sorely needed to cope up with the 5G advancement challenges. Furthermore, there has been limited research into the usefulness of multi-hop paths in establishing QoS requirements at the MAC layer [73]. The majority of the known QoS-aware directional MAC techniques [74,75] are restricted to single-hop wireless networks.

### 6.2. Control and Data Channel

Owing to the restricted penetration of mmWave-based 5G networks, control overhead for connection and mobility management is implemented on microwave communication bands, whereas data channels are established on mmWave communication bands. This complex integration has a considerable impact because numerous goals must be considered while creating a routing strategy. These objectives include enhancing the average sum rate, reducing the overall delay, and appropriately balancing the load. Thus, they are lacking in adequate research which can incorporate control channels into mmWave bands while maintaining a low overhead. Separating the control and the data channels was not necessary in the traditional 3G and 4G networks. To sum up, the traffic management of the separated channels in mmWave communication is still an open research direction, and focusing on it may pave the route for novel initial call setup and resource allocation protocols.

### 6.3. Large Scale Integration

SInce most of the previous mmWave studies were conducted before deploying 5G mmWave on a large scale in real-world settings, most resource allocation techniques in wireless communication are becoming outdated. In addition, large-scale scalability will create enormous overheads. In addition, ACK packages, which are the fundamental controls in most MAC protocols, also produce a significantly large overhead. The connected smart devices and the IoT modules also amount to large data traffic. Researchers are already concepting cloud radio access network (C-RAN) to break the base station into baseband unit (BBU) and remote radio head (RRH) and coordinating the BBUs and RRHs from a different site and the mechanism in cloud servers in next-generation 5G mmWave networks to reduce the complexity of serving hundreds of devices through the BS. Research on MAC protocols for C-RAN is still in the rudimentary stage. Consequently, it may be necessary to establish new, more robust protocols for minimizing the control overheads in large-scale cellular networks and WPANs.

### 6.4. Machine Learning Approaches

ML techniques such as deep RL (DRL) and federated learning (FL) can be implemented into the PNC to comprehend the network better when there are multiple PNCs present in a network because they highly influence the centralized WPAN. Intelligent control strategies for distributed management are challenging to establish. The novel technique of FL stands at the top of the list. FL’s purpose is to train an AI model in a distributed fashion across numerous devices, here in our case in the PNCs, utilizing local data without having to share them with other PNCs. After that, it uses central learning to decrease space and temporal complexity. However, FL is yet to be used for mmWave MAC protocols and scheduling.

Moreover, RL performs interestingly well for unknown environments, such as implementing RL-based protocol for outdoor 5G networks or indoor WLANs by estimating multiple channels or network parameters with the help of an agent. However, in dense networks, which are the primarily envisioned network architectures for mmWave enabled 5G, DRL tends to work significantly better than RL. DRL is different from the traditional supervised DL. When the state space or network matrices become complicated, the DRL is good at estimating the previous experience, actions, and states from its experience replay memory, in our case, stored in the PNC. Concurrently, our survey found that very limited resources are available for utilizing the potential of RL, and there is no study of DRL for mmWave MAC protocols, thus necessitating further investigation.

### 6.5. Multi-Hop Connectivity

To address the problems of mmWave propagation, wireless backhaul networks in the mmWave range require meshing the connection between nodes [76]. In mmWave mesh constructions, the main feature is relaying in multi-hop communications that ensure high range with LoS [10,39,77]. The LoS length is comparatively very low compared to microwave, and even though directional transmissions may overcome the increased path loss of mmWave bands, hard obstruction mediums may cause outages. To prevent outages, numerous APs must be deployed, which necessitates a wireless multi-hop architecture in situations where fiber is unavailable or extremely expensive [78]. Nonetheless, the majority of conventional state-of-the-art MAC protocols do not have the multi-hop feature.

In [15], the authors have addressed that, instead of the comprehensive multi-hop communication capability introduced in IEEE 802.11ay, existing mmWave standards offer only single or two-hop communications. The reason behind popularizing the single-hop approach might lie in the limitations of the current TDD cellular networks. This approach forces all network cells to transmit either downlink or uplink backhaul data, which is particularly inconvenient for relay nodes [79]. Such actions lead to performance bottlenecks because they are unable to utilize the two-hop link resources at their full potential. Hence, this challenge must be addressed in future research toward multi-hop compatible mmWave communication systems.

### 6.6. Cross-Layer Design

The MAC layer must respond to traffic flows from the higher layer, as well as channel variations and physical layer operations. For example, frame size per transmission period should be determined adaptively based on packet arrival from the networking layer as well as measurements from the physical layer. Measurements from the physical layer can be used to optimize transmission scheduling in the MAC layer.

### 6.7. Mobility Management

Mobility can rapidly alter the quality of established mmWave connections. Nodes in vehicle-to-everything (V2X) networks change positions at varying speeds. A management module should be created at the MAC layer to monitor node location and speed to maintain network connectivity. A mobility model for each situation must be developed for such an application. The mobility management module will determine how to operate the handover and keep the connection stable until the transmission is completed without any interruption. In mobile environments, incorporating quick switching capabilities can also help to minimize beam switching time and latency.

## 7. Conclusions

In this comparative survey, the performance requirements of the MAC layer of a mmWave wireless cellular communication system were established in terms of the capacity, spectral efficiency, and QoS. This survey focused on the adjustments necessary at the MAC layer functionalities for synchronization, random access, handover, channelization, interference management, scheduling, and association. Analysis of different protocols showed that optimizing the MAC layer can help the mmWave 5G communication system achieve near-optimal quality of experience fairness and have an advantage over the traditional PHY layer designs. This survey also provides a significant platform for academics to be motivated and to improve the outcomes of various types of challenges in next-generation MAC protocol designs for mmWave networks.
